# Correlates and Detection of Digital Health Literacy in Patients With Colorectal Carcinoma or Non-Hodgkin Lymphoma: Cross-Sectional Study

**DOI:** 10.2196/67911

**Published:** 2025-11-14

**Authors:** Thomas S Gunning, Amanda Khoudary, Osairys Billini, Andrew Ip, Lia Sorgen, John Marshall, Benjamin A Weinberg, Arnold L Potosky, Marc D Schwartz, Claire C Conley, Heather M Derry-Vick

**Affiliations:** 1 Hackensack Meridian School of Medicine, Nutley, NJ, United States; 2 Department of Internal Medicine, Montefiore Medical Center, Albert Einstein College of Medicine, Bronx, NY, United States; 3 Cancer Prevention Precision Control Institute, Center for Discovery and Innovation, Hackensack Meridian Health, 123 Metro Blvd, Nutley, NJ, 07110, United States, 1 2018803454; 4 John Theurer Cancer Center, Hackensack University Medical Center, Hackensack, NJ, United States; 5 Georgetown Lombardi Comprehensive Cancer Center, Washington, DC, United States

**Keywords:** digital health literacy, eHealth, screener, screening, colorectal carcinoma, non-Hodgkin lymphoma

## Abstract

**Background:**

Cutting-edge oncology care often depends on patients’ ability to use rapidly evolving health technology. Digital health literacy (DHL; the capacity to understand health-related information with electronic media) is an emerging, yet underexplored social determinant of health in patients with cancer.

**Objective:**

We aimed to characterize sociodemographic and clinical factors associated with DHL in patients with cancer and explore whether a single-item screener could be derived from a widely-used DHL questionnaire to detect low DHL.

**Methods:**

Patients (N=105) who received systemic treatment in the past year for colorectal carcinoma (CRC) or non-Hodgkin lymphoma (NHL) were recruited through collaborating clinics. Participants self-reported DHL using the eHealth Literacy Scale (eHEALS). They also reported general health literacy and sociodemographic and clinical characteristics. Correlations and group comparisons (independent sample *t* tests and *χ*^2^ tests, as appropriate) were used to evaluate links between DHL and sociodemographic and clinical characteristics. Receiver operating characteristic (ROC) curve analysis was used to determine whether a single eHEALS item could effectively screen for low DHL (eHEALS score ≤20).

**Results:**

Patients with a lower education level (Spearman ρ=0.29; *P*=.004) and lower general health literacy (r=0.25; *P*=.009) had lower DHL. Patients with NHL reported lower DHL than those with CRC (*t*_103_=2.72; *P*=.008). Additionally, the subset of patients who reported participation in a clinical trial (n=10) exhibited lower DHL than nonparticipants (*t*_100_=3.08; *P*=.003). Other sociodemographic and clinical characteristics were not significantly associated with DHL (all *P*>.21). The ROC curve analysis showed that eHEALS item 4 (“I know where to find helpful health resources on the Internet”) was a strong predictor of high versus low DHL (area under the curve=0.975, 95% CI 0.949‐1.00; *P*<.001).

**Conclusions:**

In this convenience sample, DHL varied based on cancer type, education level, general health literacy, and clinical trial participation. Furthermore, we found that a single item from the eHEALS has strong potential for identifying those with low DHL. These findings may inform which patients have higher need for or may benefit from DHL interventions and suggest avenues for detecting low DHL in oncology clinics.

## Introduction

Physicians, caregivers, and cancer survivors increasingly use technology as part of cancer care [1,2]. Engagement with health technologies (eg, telemedicine and patient portals) confers substantial benefits [3-5]; they facilitate access to care, increase patient engagement, and help patients manage, coordinate, and learn about their health [6-8]. However, accelerating health technology availability has increased the “digital divide” between patients who adopt health technology as part of their care and those who do not [9-11].

Digital health literacy (DHL; the capacity to find and understand health-related information with electronic media) is an emerging social determinant of health (SDOH) [12,13]. Lower DHL is associated with health risks and may impede uptake of cutting-edge patient-centered technologies aimed to optimize cancer care [14,15]. In a recent retrospective study of patients with cancer, those with higher DHL showed better overall survival than those with lower DHL [16].

Promoting equitable cancer outcomes across DHL levels calls for new methods to quickly identify low DHL among patients with cancer [17]. Accordingly, we investigated how sociodemographic and clinical factors were related to patients’ reported DHL using data from a multicenter cross-sectional study of patients with non-Hodgkin lymphoma (NHL) or colorectal carcinoma (CRC). We also examined whether a single-item screener could be derived from a widely used DHL questionnaire to maximize screening opportunities in the oncology clinic and inform future studies.

## Methods

### Study Procedures

Patients (N=105) who received systemic therapy (eg, chemotherapy or immunotherapy) within the last year for NHL or CRC were recruited between November 2022 and April 2023 from 3 clinics at MedStar Georgetown University Hospital, MedStar Washington Hospital Center, and John Theurer Cancer Center as part of a larger cross-sectional study about cancer survivorship experiences. The study’s methods were previously published, and the current analysis represents a secondary analysis of these data [18]. NHL and CRC were selected to represent both solid and hematological cancers and due to their high prevalence at collaborating clinics, frequent use of systemic therapies, and prevalence across age and gender groups (versus, eg, breast or prostate cancer) [19]. Participants reported DHL on the validated eHealth Literacy Scale (eHEALS) by rating their ability or confidence to complete digital health tasks on 8 Likert-scale items (score range: 8-40) [20,21]. Scores ≤20 indicate low DHL [22-24]. Cronbach α was 0.96 in this sample. Participants reported their age, gender, race, ethnicity, education, general health literacy (with the 3-item Health Literacy Screening questionnaire [25]), and disease- and treatment history–related clinical factors. Surveys were mailed or emailed to the patient and were completed on paper, online, or over the phone.

### Statistical Analysis

Statistical analysis used RStudio (version 2023.12.0+369) and GraphPad-Prism (version 10.3.1). We computed descriptive statistics, then used independent-sample *t* tests, *χ*^2^ tests, and correlations (as appropriate) to test associations between sociodemographic or clinical factors and DHL, with a 2-tailed α level of .05. We used simultaneous multiple linear regression to evaluate whether associations between clinical factors and DHL remained when adjusting for covariates (age, education level, and general health literacy; these were selected according to prior work or significant relationships with DHL in this dataset at *P*<.05) [13,15,26,27].

To identify how single items performed to identify low DHL, we generated receiver operating characteristic (ROC) curves for each eHEALS item using a previously described approach [28,29]. We examined the area under the curve (AUC) for each ROC curve to compare the overall performance of each item for predicting low DHL.

### Ethical Considerations

The Georgetown-MedStar Joint Oncology Institutional Review Board (STUDY00005586) approved the study. All participants provided informed consent. Study procedures included standard practices to prioritize participants’ privacy and confidentiality, including labeling surveys with participant IDs rather than identifiers, secure data storage, deidentification prior to analysis, and aggregate reporting. A US $20 gift card was offered to all participants.

## Results

### Participant Characteristics

Among the 105 participants (Table 1), 50 (48%) had NHL and 55 (52%) had CRC. Participants were primarily middle-aged to older (mean age 60, SD 13.2, range 22‐84 years), male (n=57, 54%), identified as White (n=65, 62%), and had a college-level education or higher (n=57, 54%). The mean eHEALS score was 26.9 (SD 8.24, range 8‐40), and 24% (n=25) had low DHL (score ≤20).

**Table 1.** Patient characteristics and differences between patients with colorectal carcinoma (CRC) and non-Hodgkin lymphoma (NHL) assessed in this study.

		Total (n=105)	CRC (n=55)	NHL (n=50)	*P *value
eHEALS^a^ score, mean (SD)		26.9 (8.24)	28.9 (7.91)	24.6 (8.10)	.008
Health literacy score, mean (SD)		12.6 (2.42)	12.7 (2.32)	12.5 (2.54)	.57
Age (years), mean (range)		60.3 (22-84)	57.1 (31-84)	62.9 (22-82)	.05
Female, n (%)		48 (46)	29 (53)	19 (38)	.13
Race/ethnicity^b^, (%)					<.001
	Asian, (%)	7 (7)	5 (9)	2 (4)	
	Black or African American	26 (25)	23 (42)	3 (6)	
	Hispanic or Latino	2 (2)	1 (2)	1 (2)	
	Middle Eastern or North African	4 (4)	1 (2)	3 (6)	
	White	65 (62)	25 (45)	40 (80)	
	Other or unknown	4 (4)	3 (5)	1 (2)	
Education, n (%)					.03
	High school or less	20 (19)	10 (18)	10 (20)	
	Some college	22 (21)	7 (13)	15 (30)	
	College degree	21 (20)	10 (18)	11 (22)	
	Graduate degree	36 (34)	26 (47)	10 (20)	
	Other or unknown	6 (6)	2 (4)	4 (8)	
Months from diagnosis, mean (SD)		50.9 (127.5)	36.3 (39.7)	66.9 (179.7)	.22
Treatment type, n (%)					
	Infusion therapy	99 (94)	52 (95)	47 (94)	.90
	Oral medication	62 (59)	34 (62)	28 (56)	.54
	Radiation therapy	23 (22)	21 (38)	2 (4)	<.001
Clinical trial participation, n (%)		10 (9.5)	4 (7.3)	6 (12)	.41

a eHEALS: eHealth Literacy Scale.

b Participants could select more than one option for race/ethnicity.

### Sociodemographic and Clinical Factors Associated With DHL

Those with lower education levels and lower general health literacy had lower DHL (Spearman ρ=0.29; *P*=.004 and *r*=0.25; *P*=.009, respectively). DHL did not vary by age, gender, race/ethnicity, or household income (all *P*>.21).

**Figure 1.**
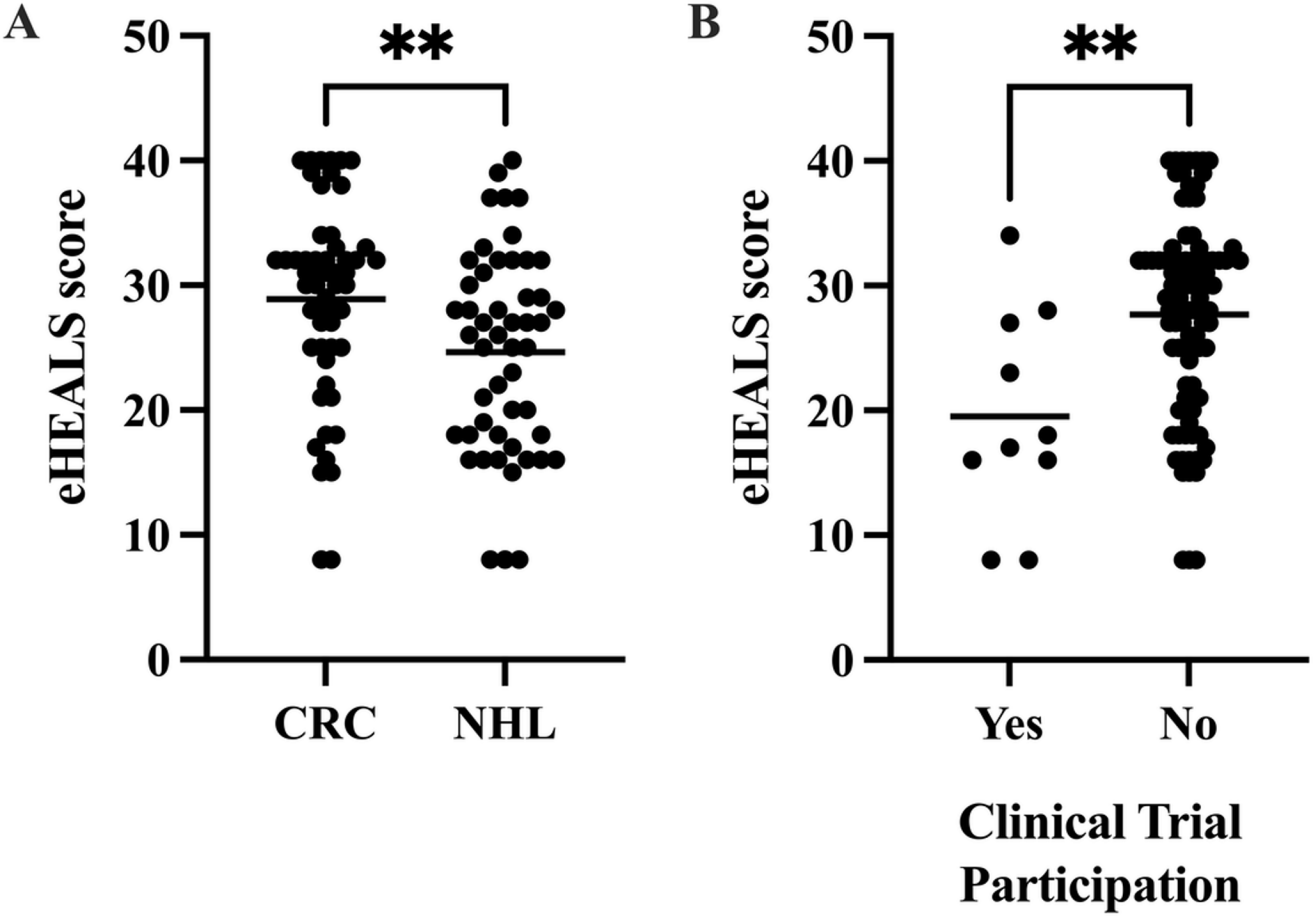
Clinical factors associated with digital health literacy (DHL) among participants. (A) Patients with colorectal carcinoma (CRC) reported higher DHL than patients with non-Hodgkin lymphoma (NHL) (*P*=.008). (B) Patients who reported participating in a clinical trial reported lower DHL than patients who did not (*P*=.003). eHEALS: eHealth Literacy Scale. **indicates *P*-value less than 0.05.

Patients with NHL reported lower DHL than patients with CRC (*t*_103_=2.72; *P*=.008; Figure 1A). This pattern persisted when controlling for age, education, and general health literacy (unstandardized b coefficient=−3.07, SE=1.65; *P*=.07). The patient subset who participated in a clinical trial (n=10) as part of their cancer treatment had lower DHL than patients who did not participate in a trial (*t*_100_=3.08; *P*=.003; Figure 1B). In the adjusted model, patients reporting trial participation still showed lower DHL when controlling for age, education, general health literacy, or disease type (b=−10.45; SE=3.20; *P*=.002; Table 2). DHL did not vary by time since diagnosis or treatment type (all *P*>.23).

**Table 2.** Multivariable linear regression model predicting eHealth Literacy Scale score as the outcome.

Variable	b	SE	*P* value
Clinical trial participation	–10.4	3.20	.002
Disease type (ref CRC^a^)	–3.20	1.60	.049
Age	–0.02	0.06	.74
Educational achievement level	1.67	0.72	.02
General health literacy	0.69	0.34	.046

a CRC: colorectal carcinoma.

### Single-Item Screener for Low DHL

**Figure 2.**
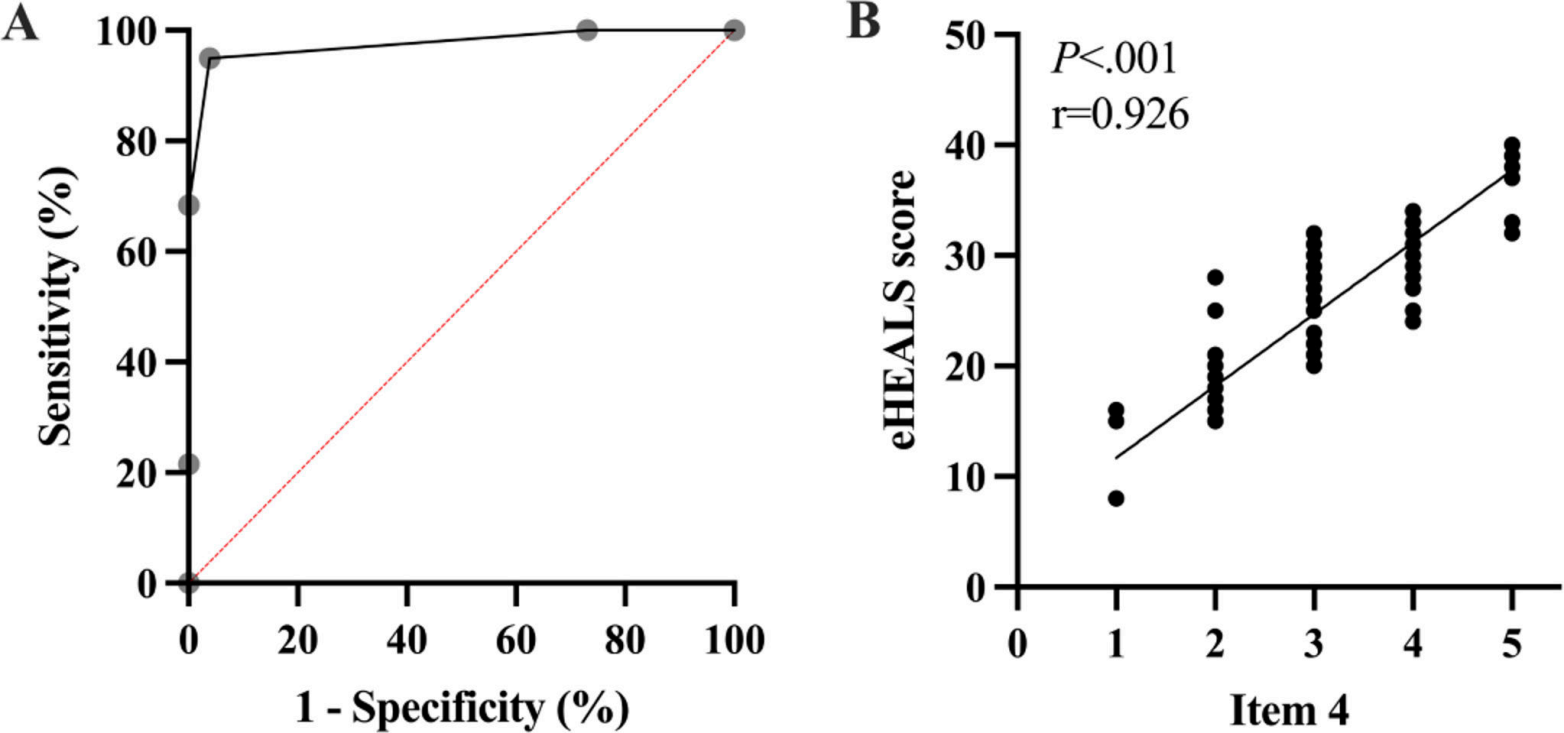
(A) Receiver operator characteristic curve for eHealth Literacy Scale (eHEALS) item 4 detects high and low digital health literacy with high sensitivity and specificity (P<.001). (B) Correlation between eHEALS item 4 and participants’ overall eHEALS scores (P<.001).

In an ROC analysis predicting low DHL (eHEALs ≤20), individual items’ AUC values ranged from 0.895 to 0.975 (Table 3, Figure S1 in Multimedia Appendix 1). eHEALS item 4, “I know where to find helpful health resources on the Internet,” had the highest AUC (0.975, 95% CI 0.949‐1.00; *P*<.001; Figure 2A). Responses to item 4 were strongly correlated to overall eHEALS scores (*r*=0.926; *P*<.001; Figure 2B). We determined the optimal threshold score for this item to predict high versus low DHL based on accuracy (Table S1 in Multimedia Appendix 1). Using the Likert-scale options (ranging 1-5), a score less than 3 predicted low DHL with an accuracy of 96.2%, a sensitivity of 94.94% (95% CI 87.69‐98.01) and a specificity of 96.15% (95% CI 81.11‐99.80).

**Table 3.** eHealth Literacy Scale (eHEALS) items’ area under the curve (AUC) values, calculated with receiver operating characteristic curve analysis. The items come from the eHEALS, developed by Norman and Skinner [20].

Item	Definition	AUC (95% CI)	*P *value
1	I know how to find helpful health resources on the Internet	0.901 (0.828‐0.974)	<.001
2	I know how to use the Internet to answer my health questions	0.939 (0.882‐0.996)	<.001
3	I know what health resources are available on the Internet	0.953 (0.904‐1.00)	<.001
4	I know where to find helpful health resources on the Internet	0.975 (0.949‐1.00)	<.001
5	I know how to use the health information I find on the Internet to help me	0.935 (0.873‐0.996)	<.001
6	I have the skills I need to evaluate the health resources I find on the Internet	0.940 (0.890‐0.990)	<.001
7	I can tell high quality from low quality health resources on the Internet	0.949 (0.910‐0.988)	<.001
8	I feel confident in using information from the Internet to make health decisions	0.895 (0.831‐0.959)	<.001

## Discussion

### Principal Findings

DHL allows patients to benefit from technological advances in oncology care. However, little is known about which patients with cancer are more likely to experience low DHL, and clinically feasible ways to identify patients with low DHL are limited. Here, we examined sociodemographic and clinical factors associated with DHL and explored whether single items could identify patients with low DHL. Patients with a lower education level and general health literacy had lower DHL. Patients with NHL also had lower DHL than those with CRC. Interestingly, those who reported participating in a clinical trial had lower DHL than those who did not. ROC analysis showed that agreement with a single item from the widely used eHEALS (“I know where to find helpful health resources on the Internet”) was strongly predictive of low DHL; this may be a brief yet sensitive way to screen for low DHL.

Links between DHL and sociodemographic factors have previously been reported [13,15,26,27], but clinical correlates have been less frequently explored. We observed notable differences in DHL between patients with CRC and NHL that remained after adjusting for covariates. Future studies should identify drivers of DHL differences by cancer type in larger samples. We observed a novel, unexpected association between lower DHL and clinical trial participation, contrasting with prior findings that clinical trial participants used the internet first, before physicians, for clinical trial information [30]. On the other hand, it is possible that patients with worse DHL have poorer outcomes, thus necessitating later lines of therapy and enrollment in clinical trials, which could help to explain our findings. The complex relationship between clinical trial participation and DHL requires further investigation in prospective studies with electronic medical record–documented trial participation.

Rapidly and effectively identifying those with low DHL is critical to advancing this research area. Short-form screeners are well-suited for time-limited oncology clinics [29,31]. Our findings indicate that disagreeing or strongly disagreeing with the statement “I know where to find helpful health resources on the Internet” is a candidate screening criterion for low DHL. If validated in larger samples and other cancers, the item could be included in previsit paperwork, and identified patients with low DHL could receive additional support (eg, for telehealth and patient portals) or alternative modalities (eg, print resources). Future studies could also use this item to quickly screen for digital health needs in study candidates.

### Limitations

This study’s small convenience sample may limit its statistical power to identify associations and may restrict generalizability. Although we included people with solid and hematological cancers, correlates and best screening items for DHL may differ across other cancer types. Nonetheless, the observed links to sociodemographic factors are consistent with prior literature, and the full range of possible eHEALS scores (8-40) was represented in our sample. Future studies should include larger samples, more clinical trial participants, and more diverse sociodemographic and cancer groups. Additionally, future studies should include family members and other care partners, who have an important role in helping patients with lower DHL navigate their care.

### Conclusions

DHL is an emerging and actionable SDOH impacting health outcomes for people with cancer. These results provide insight into how DHL varies across cancer patients and suggest a highly predictive single-item screener that may be used to identify patients with low DHL. Using this item, if it is further validated, could increase opportunities for clinical support and developing DHL interventions better suited to patient needs.

## Supplementary material

10.2196/67911 Multimedia Appendix 1 Receiver operating characteristic curves for each eHealth Literacy Scale item; sensitivity and specificity for item 4.

## Abbreviations

AUC: area under the curve
CRC: colorectal carcinoma
DHL: digital health literacy
eHEALS: eHealth Literacy Scale
NHL: non-Hodgkin lymphoma
ROC: receiver operator characteristic
SDOH: social determinant of health
