# Supplementary material for: Correlates and Detection of Digital Health Literacy in Patients With Colorectal Carcinoma or Non-Hodgkin Lymphoma: Cross-Sectional Study
Source: JMIR Cancer. 2025 Nov 14;11:e67911. doi: 10.2196/67911 (PMC12617828; doi:10.2196/67911)
Supplement: Multimedia Appendix 1 [file cancer-v11-e67911-s001.docx]

**Figure S1**. ROC curves for each item of the eHEALS questionnaire.


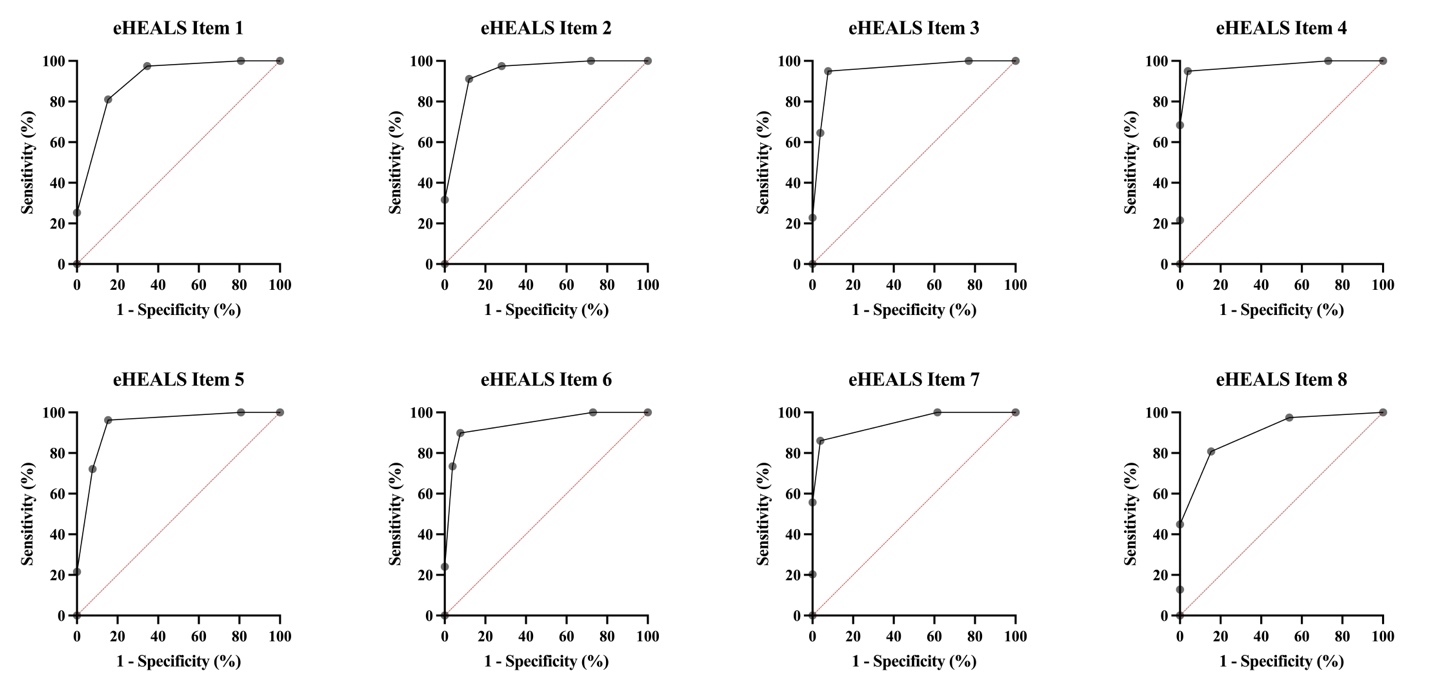


**Table S1**. Sensitivities and specificities of ROC curve for detecting low DHL using eHEALS item 4.

| Threshold | Sensitivity  (%) | 95% CI | Specificity  (%) | 95% CI | Accuracy  (%) |
| --- | --- | --- | --- | --- | --- |
| <2 | 100.0 | 95.36% to 100.0% | 26.92 | 13.70% to 46.08% | 81.90 |
| <3 | 94.94 | 87.69% to 98.01% | 96.15 | 81.11% to 99.80% | 96.19 |
| <4 | 68.35 | 57.45% to 77.55% | 100.0 | 87.13% to 100.0% | 75.24 |
| <5 | 21.52 | 13.89% to 31.79% | 100.0 | 87.13% to 100.0% | 40.95 |
